# Supplementary material for: Brominated Flame Retardants in Children’s Room: Concentration, Composition, and Health Risk Assessment
Source: Int J Environ Res Public Health. 2021 Jun 14;18(12):6421. doi: 10.3390/ijerph18126421 (PMC8296256; doi:10.3390/ijerph18126421)
Supplement: Supplementary file 1 [file ijerph-18-06421-s001.zip › ijerph-1226345-supplementary.pdf]

International Journal of
Environmental Research and Public Health

MDPI

Supplementary Information

Brominated Flame Retardants in Children's Room: Concentration, Composition, and Health Risk Assessment

Douha Bannan [1], Nadeem Ali [2,*], Nabil A. Alhakamy [3], Mohamed A. Alfaleh [4], Waleed S. Alharbi [3], Muhammad Imtiaz Rashid [2], Nisreen Rajeh [5] and Govindan Malarvannan [6,*]

[1] Faculty of Pharmacy, Pharmacy Practice, King Abdulaziz University, Jeddah 21589, Saudi Arabia; Dbannan@kau.edu.sa
[2] Center of Excellence in Environmental Studies, King Abdulaziz University, Jeddah 21589, Saudi Arabia; irmaliks@gmail.com
[3] Faculty of Pharmacy, Pharmaceutics Department, King Abdulaziz University, Jeddah 21589, Saudi Arabia; nalhakamy@kau.edu.sa (N.A.A.); wsmalharbi@kau.edu.sa (W.S.A.)
[4] Natural Products and Alternative Medicine Department, Faculty of Pharmacy, King Abdulaziz University, Jeddah 21589, Saudi Arabia; maalfaleh@kau.edu.sa
[5] Department of Anatomy, Medical College, King Abdulaziz University, Jeddah 21589, Saudi Arabia; nrajeh@kau.edu.sa
[6] Toxicological Center, University of Antwerp, Wilrijk 2610, Belgium
* Correspondence: nabahadar@kau.edu.sa (N.A.); malarvannan.govindan@uantwerpen.be (G.M.)

Table S1. Important parameters collected on the questionnaire during sample collection.

Parameters	A	B	C	D	E	F	G	H	I	J	K	L	M	N	O	P	Q	R	S	T
1	2	16	1	4-6	AC	W	12h	D	N	Y	90%	10%	60%	20%	0%	20%	0%	0%	N	E
2	1	20	1	12	AC	W	15h	D	Y	Y	100%	0%	85%	5%	5%	5%	0%	0%	R, A	E
3	1	16	1	10	AC/W	S	12h	ID	Y	Y	90%	10%	70%	5%	5%	0%	10%	0%	N	E
4	5	16	2	7-11	AC	W	12h	D	Y	N	60%	40%	30%	30%	40%	0%	0%	0%	N	E
5	2	12	2	2-6	AC	W	10h	D	N	Y	80%	20%	60%	10%	10%	10%	10%	0%	N	E
6	2	20	2	8-6	AC	W	16h	D	N	Y	80%	20%	50%	20%	5%	10%	0%	15%	N	E
7	3	12	3	7-9-11	AC/W	W	14h	ID	N	Y	100%	0%	0%	0%	0%	0%	100%	0%	R	NE
8	4	16	2	3-6	AC	W	6h	ID	Y	N	90%	10%	50%	5%	10%	30%	0%	5%	N	E
9	2	15.8	2	5-7	AC	W	16h	ID	N	Y	100%	0%	80%	5%	0%	10%	2%	3%	A	E
10	2	24	4	6-10-11-13	AC	S	15h	D	N	Y	50%	50%	64%	4%	0%	30%	1%	1%	A, S	E
11	4	12	1	5	AC	S	10h	D	N	N	80%	20%	20%	50%	1%	28%	0%	1%	N	MTE
12	2	24	2	4-2	AC	S	24h	D	N	N	70%	30%	40%	30%	0%	30%	0%	0%	A	E
13	2	12	2	9-6	AC	W	5h	ID	Y	Y	50%	50%	80%	0%	0%	20%	0%	0%	N	E
14	2	16	1	5	AC	W	10h	D	N	N	90%	10%	10%	20%	5%	40%	5%	20%	N	E
15	3	12	4	4-5-7-9	AC	W	12h	D	Y	Y	50%	50%	60%	5%	10%	10%	5%	10%	N	E
16	3	16	2	8-12	AC	W	12h	ID	N	N	0%	0%	0%	0%	0%	0%	100%	0%	N	E
17	7	16	2	5-8	AC	W	15h	ID	N	N	100%	0%	80%	5%	0%	10%	2%	3%	N	E
18	3	15.8	3	5-7-9	AC	W	16h	D	N	Y	90%	10%	10%	20%	5%	40%	5%	20%	N	E
19	4	32	1	6	AC	S	14h	ID	Y	Y	5%	95%	60%	20%	5%	20%	1%	0%	N	E
20	2	16	2	4-6	AC	W	10h	D	N	N	30%	70%	80%	5%	0%	15%	0%	0%	N	E
21	3	12.3	4	4-6-8-10	AC	W	12	D	Y	N	30%	70%	40%	0%	0%	50%	0%	10%	N	E
22	3	13.4	2	2-4	AC	W	6h	D	Y	N	70%	30%	50%	0%	20%	30%	0%	0%	R	E
23	4	16	2	4-6	AC	W	10h	ID	N	Y	60%	40%	70%	5%	0%	25%	0%	0%	N	E
24	7	28	3	3-3-6	AC	S	8h	D	Y	Y	80%	20%	40%	35%	10%	5%	5%	5%	A	E
25	3	12	3	12-7-3	AC	W	16h	D	Y	Y	70%	30%	20%	15%	5%	40%	10%	10%	N	E
26	7	16	1	7	AC	C	14h	ID	Y	N	100%	0%	20%	0%	20%	60%	0%	0%	A	E
27	4	13	3	7	AC	C	10h	D	Y	N	95%	5%							R	E
28	3	16	2	8-10	AC/W	W	10h	D	N	N	70%	30%	40%	30%	0%	30%	0%	0%	A	E
29	3	13.4	2	5-9	AC	W	12h	ID	Y	Y	90%	10%	10%	20%	5%	40%	5%	20%	N	E
30	3	16	3	6-8-12	AC	W	15h	D	N	Y	70%	30%	50%	0%	20%	30%	0%	0%	N	E

A- Number of dusting per week; B- Size of room (m3); C- Number of kids sharing room; D- Age of kids sharing a room (years); E- Room ventilation (Air conditioning (AC), window (W)); F- AC type (window (W), split (S), centralized (C)); G- AC turned on the day (hours (h)); H- Cross ventilation with outside (Direct (D), indirect (ID)); I- Furniture with upholstery foam (no (N), yes (Y)); J- PC, TV, and other electronics (no (N), yes (Y)); K- Toys (older than one year) % of the total toys; L- Toys (new) % of the whole toys; M- Hard plastic toys; N- Soft plastic toys; O- Wooden toys; P- Stuff toys; Q- Electronic toys; R- Others; S- Any health issues such as respiratory (R), allergy (A), skin problems (S), and late learning (LL) etc., among children on consistent bases; T- Family resourcefulness (income) (not enough (NE), enough (E), more than enough (MTE)).

Table S2. Comparing median levels of BFRs data from different countries for indoor dust (ng/g) and indoor air (pg/m^3).

Country	Sampling site	BDE 28	BDE 47	BDE 99	BDE 100	BDE 153	BDE 183	BDE 209	TBB	TBPH	BTBPE
					Indoor Dust						
Romania[1]	Homes (n=47)	0.10	2	0.70	0.30	0.80	0	275	<dl	10	4
Sweden[2]	Homes (n=27)	0.99	21	17	2.6	1.9	0	310	2.6	61	6.3
Portugal[3]	Homes (n=28)	<0.2	5.7	6.3	1.2	0.75	2.4	270			1.2
Germany[4]	Homes (n=20)	0.1	5.7	9.2	1.6	2.1	9.3	950	<dl	343	<dl
Norway[5]	Homes (n=48)	0.688	126	171	33	26	3.22	325	2.54	79	3.76
Poland[6]	Homes (n=12)	3.8	5.4	1.4	<dl	<dl	3.9	219			
Turkey[7]	Homes (n=4)	0.12	62	34	2.4	26	21	574	184	0.13	108
Spain[8]	Homes (n=5)	<dl	7.3	5.9	<dl	7.9	33.4	3526			
USA[9]	Pre-school (n=39)	<dl	769	1031	212	125	17	1443			
[10]USA	Homes (n=30)	0.77	452	741	99	41	1.0	1720			
[11]USA	Homes (n=59)								337	186	22.3
Australia[12]	Schools (n=28)	1	40	91	0	12	4.7	217			
South Korea[13]	Schools (n=24)	2.8[a]	222[a]	216[a]	68[a]	30[a]	18[a]	4195[a]			
	Academies (15)	7.6[a]	46[a]	42[a]	6.9[a]	33[a]	237[a]	6267[a]			
	Homes (n=12)	0.8[a]	3.2[a]	14[a]	0.8[a]	219[a]	26[a]	4354[a]			
China[14]	Homes (n=23)	38	102	75	85	11	78	975			
Japan[15]	Schools (n=18)	2.54	8.9	7.89	1.63	2.82	13.2	995			
	Homes (n=10)	0.64	5.65	6.11	1.3	15	71	1429			
Taiwan[16]	Urban school (n=6)	0.14	2.87	5.66	0.99	2.59	8.25	263			
Pakistan[17]	Homes (n=15)	<0.1	1.3	1.7	0.3	0.6	1.5	140	0.4	5.8	15
Kuwait[17]	Home (n=15)	0.4	9.5	12	2.3	2.4	1.9	310	6.6	54	6.8
Egypt[18]	Homes (n=17)	0.34	1.69	2.7	0.37	6.26	1.05	40	0.81	0.12	0.24
Iraq[19]	Homes (n=18)	<dl	3.6	6.67	0.6	0.61	7.5	612	5.3	64	14
Saudi Arabia[20]	Homes (n=15)	<dl	27	35	5	4	4	275	16	25	5
Current study	Children rooms (n=30)	<dl	2	<dl	<dl	<dl	<dl	3150	3	7	2
					Indoor Air						
Kuwait[21]	Home (n=46)	0.4	3.6	2.4	0.5	0.2	0.2				
USA[22]	Home LR (n=20)	25	145	60	12	3.5	94				
Sweden [23]	Apartment (n=44)	2.2	11	2.7		0.74	1.3	24			
	Day care centers (n=10)	3.4	110	26		1.2	6.6	1100			
China[24]	Home (n=60)	3.21	35	14	9.66	10	4.5	97			
Norway[25]	Home (n=47)	7.53	128	21	6.78	0.927	<dl	3.76	<dl	<dl	<dl
South Korea[13]	Schools (n=54)	5[a]	399[a]	279[a]	135[a]	8[a]	15[a]	208[a]			
	Academies (31)	2[a]	17[a]	6[a]	2[a]	11[a]	3[a]	148[a]			
	Homes (n=12)	4[a]	33[a]	18[a]	6[a]	11[a]	4[a]	412[a]			
Taiwan[25]	Home (n=3)	7.8[a]	4.97[a]	0.74[a]	0.14[a]	0.66[a]	1.00[a]	57[a]			

a= Mean value; <dl = Detection limit.

Table S3. Different exposure scenarios of estimated daily exposure *via* indoor dust ingestion (ng/kg/bw/day) and PM10 (pg/kg/bw/day) to BFRs for Saudi young children from their rooms.

Analytes	RfD	Low dust intake						High dust intake						Air Inhalation					
		12 kg BW		25 kg BW		40 kg BW		12 kg BW		25 kg BW		40 kg BW		12 kg BW		25 kg BW		40 kg BW	
		90th %ile	Mean	90th %ile	Mean	90th %ile	Mean	90th %ile	Mean	90th %ile	Mean	90th %ile	Mean	90th %ile	Mean	90th %ile	Mean	90th %ile	Mean
**BDE 28**	100	0.21	0.07	0.10	0.03	0.06	0.02	0.83	0.29	0.40	0.14	0.25	0.09	0.03	0.00	0.02	0.01	0.02	0.01
**BDE 47**	100	0.33	0.28	0.16	0.13	0.10	0.08	1.31	1.10	0.63	0.53	0.39	0.33	0.00	0.00	0.04	0.03	0.03	0.03

**BDE100**	100	0.04	0.08	0.02	0.04	0.01	0.02	0.16	0.33	0.08	0.16	0.05	0.10	0.00	0.00	0.00	0.01	0.00	0.01
**BDE 99**	100	0.94	0.61	0.45	0.29	0.28	0.18	3.74	2.45	1.80	1.18	1.12	0.73	0.00	0.00	0.11	0.07	0.09	0.06
**BDE153**	200	0.63	0.40	0.30	0.19	0.19	0.12	2.51	1.61	1.20	0.77	0.75	0.48	0.00	0.00	0.07	0.05	0.06	0.04
**BDE154**	200	0.03	0.04	0.01	0.02	0.01	0.01	0.11	0.15	0.05	0.07	0.03	0.05	0.01	0.00	0.00	0.00	0.00	0.00
**BDE183**	300	0.01	0.11	0.00	0.05	0.00	0.03	0.04	0.43	0.02	0.21	0.01	0.13	0.02	0.01	0.00	0.01	0.00	0.01
**BDE209**	7000	72	30.3	34.5	14.5	21.7	9.1	287	121	138	58.4	86.3	36.3	0.19	0.09	8.25	3.48	6.54	2.76
**TBB**	20000	2.97	10.4	1.42	5.01	0.89	3.13	11.9	41.7	5.69	20.1	3.56	12.5	0.01	0.07	0.34	1.20	0.27	0.95
**BTBPE**	230000	0.06	0.07	0.03	0.03	0.02	0.02	0.24	0.28	0.12	0.14	0.07	0.08	0.04	0.02	0.01	0.01	0.01	0.01
**TBPH**	20000	5.83	2.41	2.80	1.15	1.75	0.72	23.3	9.62	11.2	4.62	6.99	2.89	0.01	0.00	0.67	0.28	0.53	0.22

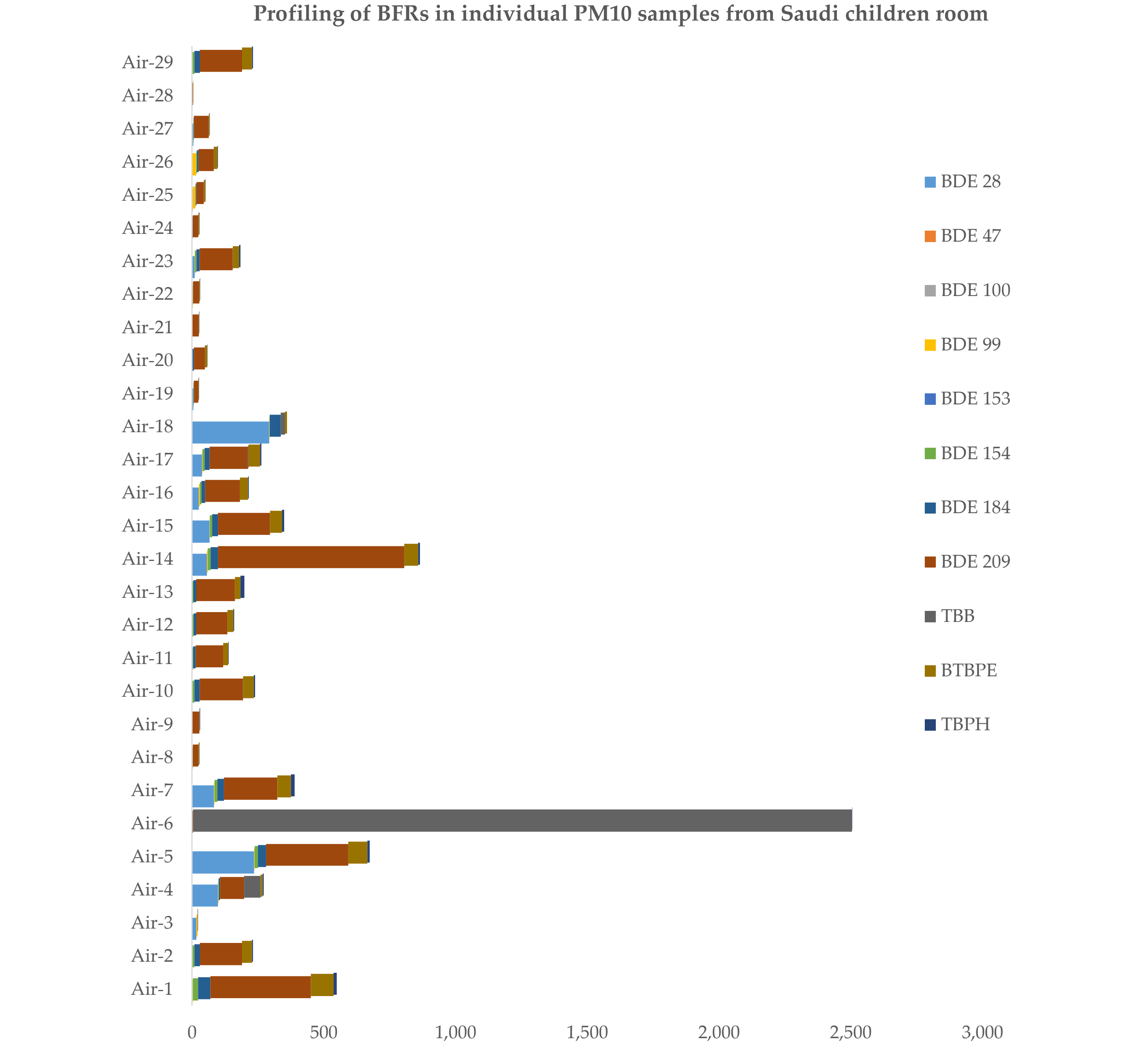

Figure S1. Profiling of analyzed BFRs in individual PM10 samples from Saudi children rooms. Values on the longitudinal axis are in pg/m^3.

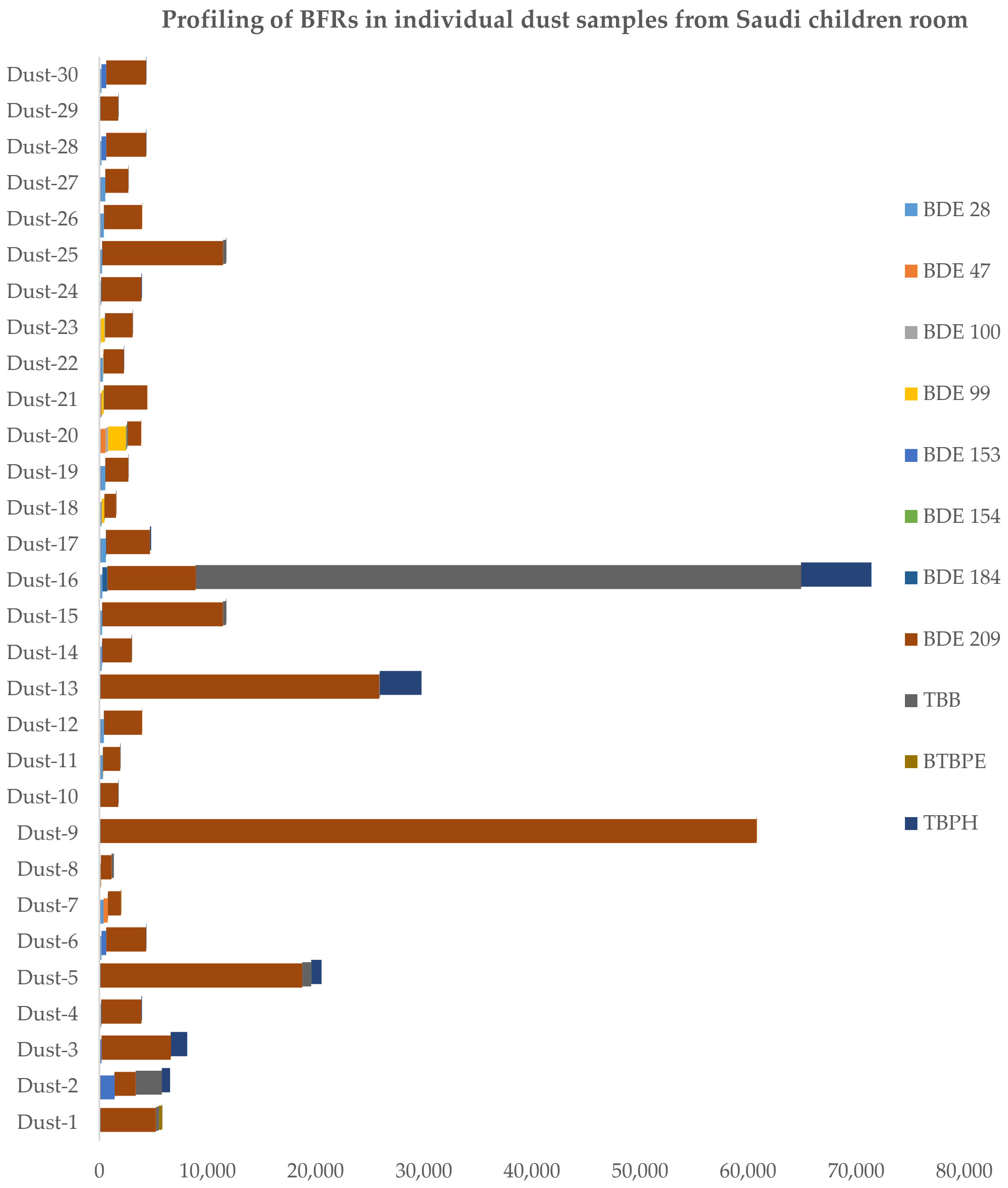

Figure S2. Profiling of analyzed BFRs in individual dust samples from Saudi children rooms. Values on the longitudinal axis are in ng/g of dust.

References

1. Dirtu, A.C., Ali, N., Van den Eede, N., Neels, H. and Covaci, A., 2012. Country specific comparison for profile of chlorinated, brominated and phosphate organic contaminants in indoor dust. Case study for Eastern Romania, 2010. *Environment international*, *49*, pp.1-8.
2. Sahlström, L.M., Sellström, U., de Wit, C.A., Lignell, S. and Darnerud, P.O., 2015. Estimated intakes of brominated flame retardants via diet and dust compared to internal concentrations in a Swedish mother–toddler cohort. *International Journal of Hygiene and Environmental Health*, *218*(4), pp.422-432.
3. Coelho, S.D., Sousa, A.C., Isobe, T., Kim, J.W., Kunisue, T., Nogueira, A.J. and Tanabe, S., 2016. Brominated, chlorinated and phosphate organic contaminants in house dust from Portugal. *Science of The Total Environment*, *569*, pp.442-449.
4. Fromme, H., Hilger, B., Kopp, E., Miserok, M. and Völkel, W., 2014. Polybrominated diphenyl ethers (PBDEs), hexabromocyclododecane (HBCD) and "novel" brominated flame retardants in house dust in Germany. *Environment international*, *64*, pp.61-68.
5. Cequier, E., Ionas, A.C., Covaci, A., Marcé, R.M., Becher, G. and Thomsen, C., 2014. Occurrence of a broad range of legacy and emerging flame retardants in indoor environments in Norway. *Environmental science & technology*, *48*(12), pp.6827-6835.
6. Król, S., Namieśnik, J. and Zabiegała, B., 2014. Occurrence and levels of polybrominated diphenyl ethers (PBDEs) in house dust and hair samples from Northern Poland; an assessment of human exposure. *Chemosphere*, *110*, pp.91-96.
7. Kurt-Karakus, P.B., Alegria, H., Jantunen, L., Birgul, A., Topcu, A., Jones, K.C. and Turgut, C., 2017. Polybrominated diphenyl ethers (PBDEs) and alternative flame retardants (NFRs) in indoor and outdoor air and indoor dust from Istanbul-Turkey: levels and an assessment of human exposure. *Atmospheric Pollution Research*, *8*(5), pp.801-815.
8. Cristale, J., Hurtado, A., Gómez-Canela, C. and Lacorte, S., 2016. Occurrence and sources of brominated and organophosphorus flame retardants in dust from different indoor environments in Barcelona, Spain. *Environmental research*, *149*, pp.66-76.
9. Darrow, L.A., Jacobson, M.H., Preston, E.V., Lee, G.E., Panuwet, P., Hunter Jr, R.E., Marder, M.E., Marcus, M. and Barr, D.B., 2017. Predictors of serum polybrominated diphenyl ether (PBDE) concentrations among children aged 1–5 years. *Environmental science & technology*, *51*(1), pp.645-654.
10. Stapleton, H.M., Misenheimer, J., Hoffman, K. and Webster, T.F., 2014. Flame retardant associations between children's handwipes and house dust. *Chemosphere*, *116*, pp.54-60.
11. Brown, F.R., Whitehead, T.P., Park, J.S., Metayer, C. and Petreas, M.X., 2014. Levels of non-polybrominated diphenyl ether brominated flame retardants in residential house dust samples and fire station dust samples in California. *Environmental research*, *135*, pp.9-14.
12. Toms, L.M.L., Mazaheri, M., Brommer, S., Clifford, S., Drage, D., Mueller, J.F., Thai, P., Harrad, S., Morawska, L. and Harden, F.A., 2015. Polybrominated diphenyl ethers (PBDEs) in dust from primary schools in South East Queensland, Australia. *Environmental research*, *142*, pp.135-140.
13. Lim, Y.W., Kim, H.H., Lee, C.S., Shin, D.C., Chang, Y.S. and Yang, J.Y., 2014. Exposure assessment and health risk of polybrominated diphenyl ether (PBDE) flame retardants in the indoor environment of elementary school students in Korea. *Science of the total environment*, *470*, pp.1376-1389.
14. Kang, Y., Wang, H.S., Cheung, K.C. and Wong, M.H., 2011. Polybrominated diphenyl ethers (PBDEs) in indoor dust and human hair. *Atmospheric Environment*, *45*(14), pp.2386-2393.
15. Mizouchi, S., Ichiba, M., Takigami, H., Kajiwara, N., Takamuku, T., Miyajima, T., Kodama, H., Someya, T. and Ueno, D., 2015. Exposure assessment of organophosphorus and organobromine flame retardants via indoor dust from elementary schools and domestic houses. *Chemosphere*, *123*, pp.17-25.
16. Gou, Y.Y., Que, D.E., Chuang, C.Y., Chao, H.R., Shy, C.G., Hsu, Y.C., Lin, C.W., Chuang, K.P., Tsai, C.C. and Tayo, L.L., 2016. Dust levels of polybrominated diphenyl ethers (PBDEs) and polybrominated dibenzo-p-dioxins/furans (PBDD/Fs) in the Taiwanese elementary school classrooms: Assessment of the risk to school-age children. *Science of The Total Environment*, *572*, pp.734-741.
17. Ali, N., Ali, L., Mehdi, T., Dirtu, A.C., Al-Shammari, F., Neels, H. and Covaci, A., 2013. Levels and profiles of organochlorines and flame retardants in car and house dust from Kuwait and Pakistan: implication for human exposure via dust ingestion. *Environment international*, *55*, pp.62-70.
18. Hassan, Y. and Shoeib, T., 2015. Levels of polybrominated diphenyl ethers and novel flame retardants in microenvironment dust from Egypt: an assessment of human exposure. *Science of the Total Environment*, *505*, pp.47-55.
19. Al-Omran, L.S. and Harrad, S., 2016. Distribution pattern of legacy and "novel" brominated flame retardants in different particle size fractions of indoor dust in Birmingham, United Kingdom. *Chemosphere*, *157*, pp.124-131.
20. Ali, N., Eqani, S.A.M.A.S., Ismail, I.M.I., Malarvannan, G., Kadi, M.W., Albar, H.M.S., Rehan, M. and Covaci, A., 2016. Brominated and organophosphate flame retardants in indoor dust of Jeddah, Kingdom of Saudi Arabia: implications for human exposure. *Science of the Total Environment*, *569*, pp.269-277.
21. Gevao, B., Al-Bahloul, M., Al-Ghadban, A.N., Ali, L., Al-Omair, A., Helaleh, M., Al-Matrouk, K. and Zafar, J., 2006. Polybrominated diphenyl ethers in indoor air in Kuwait: Implications for human exposure. *Atmospheric Environment*, *40*(8), pp.1419-1426.

22. Allen, J.G., McClean, M.D., Stapleton, H.M., Nelson, J.W. and Webster, T.F., 2007. Personal exposure to polybrominated diphenyl ethers (PBDEs) in residential indoor air. *Environmental science & technology*, *41*(13), pp.4574-4579.
23. de Wit, C.A., Björklund, J.A. and Thuresson, K., 2012. Tri-decabrominated diphenyl ethers and hexabromocyclododecane in indoor air and dust from Stockholm microenvironments 2: indoor sources and human exposure. *Environment international*, *39*(1), pp.141-147.
24. Wang, W., Zheng, J., Chan, C.Y., Huang, M.J., Cheung, K.C. and Wong, M.H., 2014. Health risk assessment of exposure to polybrominated diphenyl ethers (PBDEs) contained in residential air particulate and dust in Guangzhou and Hong Kong. *Atmospheric Environment*, *89*, pp.786-796.
25. Shy, C.G., Hsu, Y.C., Shih, S.I., Chuang, K.P., Lin, C.W., Wu, C.W., Chuang, C.Y. and Chao, H.R., 2015. Indoor level of polybrominated diphenyl ethers in the home environment and assessment of human health risks. *Aerosol and Air Quality Research*, *15*(4), pp.1494-1505.
